# Case series of *de novo* occurrence of Graves’ disease following thermal ablation for benign thyroid nodules: an uncommon event that should be considered

**DOI:** 10.1530/ETJ-25-0205

**Published:** 2025-12-23

**Authors:** Marsida Teliti, Beatrice Grillini, Isabella Chiardi, Flavia Magri, Francesca Coperchini, Laura Croce, Mario Rotondi, Spyridon Chytiris

**Affiliations:** ^1^ Department of Internal Medicine and Therapeutics, University of Pavia, Pavia, Italy; ^2^ Unit of Endocrinology and Metabolism, Laboratory for Endocrine Disruptors, Istituti Clinici Scientifici Maugeri IRCCS, Pavia, Italy

**Keywords:** Graves’ disease, hyperthyroidism, thermal ablation, thyroid nodule, thyroid autoimmunity

## Abstract

**Introduction:**

Thermal ablation (TA), including radiofrequency (RFA) and microwave ablation (MWA), is a widely used, minimally invasive alternative to surgery for benign thyroid nodules. While transient, self-limited thyrotoxicosis is a recognized effect of the procedure, the onset of Graves’ disease (GD) post-TA remains exceedingly rare and poorly characterized.

**Case reports:**

We report three cases of new-onset GD occurring 3–10 months after RFA or MWA, identified among more than 500 patients treated at our tertiary care Endocrinology Unit. All patients were female and had undergone TA for cytologically benign nodules. Two had a documented history of thyroid autoimmunity. At diagnosis, all three exhibited suppressed TSH, elevated thyroid hormone levels, and positive anti-TSH receptor antibodies (TRAb). None had been tested for TRAb before treatment. One case occurred during pregnancy and was managed with propylthiouracil; the others received antithyroid treatment with methimazole.

**Conclusion:**

Although rare, GD may develop following TA, likely through immune activation triggered by thyroid tissue injury in genetically predisposed individuals. Routine TRAb screening before TA is not currently justified; however, clinical awareness is essential to distinguish transient post-ablation thyrotoxicosis from true GD and to ensure timely initiation of antithyroid therapy when appropriate. Despite these rare cases, the overall incidence remains very low, confirming the excellent safety profile of TA for benign thyroid nodules.

## Established facts

- Transient thyrotoxicosis is a known and relatively common consequence of TA due to hormone release from injured thyroid tissue.
- The safety profile of TA is well established, with rare and mostly self-limited endocrine complications.

## Novel insights

- This case series is among the first to report multiple new-onset GD cases following TA for benign thyroid nodules.
- Thyroid tissue injury from RFA or MWA may act as a trigger for autoimmune activation in predisposed individuals.
- Distinguishing transient post-ablation thyrotoxicosis from true Graves’ disease is clinically relevant to ensure timely initiation of antithyroid therapy when appropriate.

## Introduction

Thermal ablation (TA) has emerged as a widely accepted, minimally invasive alternative to surgery for the management of benign thyroid nodules. According to the European Thyroid Association (ETA) guidelines (1), TA is generally well tolerated and offers relevant therapeutic benefits. Radiofrequency ablation (RFA) and microwave ablation (MWA) are considered safe and effective procedures, achieving substantial nodule volume reduction in most cases. However, TA may be associated with complications, which include voice changes due to transient recurrent laryngeal nerve injury, nodule rupture (usually managed conservatively), hematoma (often mild and self-limiting), nausea and vomiting (typically related to sedative use), and superficial skin burns (2). In some patients, TA may also induce a transient phase of thyrotoxicosis caused by the acute release of thyroid hormones (T3 and T4) into the circulation (3). This condition is typically self-limiting, with spontaneous resolution within a few weeks. Conversely, the development of Graves’ disease (GD) following TA has been rarely reported. GD is an autoimmune disorder characterized by the presence of stimulating autoantibodies targeting the thyroid-stimulating hormone receptor (TSHR), leading to hyperthyroidism. To date, only isolated case reports and a few incidental cases within large series have described this phenomenon, warranting further investigation (4, 5, 6, 7).

In the present study, we describe three cases of new-onset GD that developed after RFA or MWA for cytologically benign thyroid nodules. We outline the clinical course, diagnostic features, and therapeutic management of each case and discuss possible underlying mechanisms based on a review of the existing literature.

## Case series

### Patient 1

A 50-year-old female was referred to the Endocrinology Unit for evaluation of a palpable thyroid nodule. Neck ultrasound revealed a total thyroid volume of 20 mL. The left lobe was enlarged and almost entirely occupied by a hypoechoic solid nodule measuring 37 × 33 × 19 mm (estimated volume: 12 mL) characterized by intra- and peri-nodular vascularity on color Doppler. The right lobe was regular in size, with homogeneous echostructure, and the presence of a solid, hypoechoic nodule measuring 10 mm in diameter in the para-isthmic region. The isthmus was of regular thickness.

Biochemical testing showed thyroid-stimulating hormone (TSH) at 0.68 µUI/mL, negative calcitonin, and negative thyroglobulin (TgAb) and thyroid peroxidase (TPOAb) antibodies. TRAb were not assessed. Fine-needle aspiration cytology (FNAC) confirmed a benign lesion (Bethesda II/TIR2). No cytological features suggestive of preexisting thyroid autoimmunity were observed. Due to compressive symptoms, the patient was offered RFA.

The patient underwent RFA. One month later, ultrasound showed a volume reduction rate (VRR) in nodule volume of 48% (estimated volume: 6.23 mL), with normal vascularization of the thyroid. The patient was asymptomatic, and no biochemical reassessment was performed at that time.

At 6 months, further volume reduction to 4.4 mL was observed (VRR: 63%), along with absent vascularization. Blood tests showed suppressed TSH (<0.005 µUI/mL) and mildly elevated FT4 (17.9 ng/L; normal range (NR): 9.3–17), without recent iodine exposure. Transient thyrotoxicosis was suspected, and antithyroid therapy was not initiated.

Eight months after RFA, the patient complained of tachycardia. Biochemical evaluation confirmed overt hyperthyroidism with suppressed TSH, markedly elevated FT4 and FT3, and TRAb at 7.6 U/L, consistent with new-onset GD. Methimazole (MMI) 20 mg/day was initiated. No signs of thyroid-associated orbitopathy were present.

After 6 weeks of therapy, TSH remained suppressed, FT4 normalized, FT3 remained elevated, and TRAb persisted at 7.2 U/L.

At the most recent follow-up, 2 years after RFA, the patient was euthyroid under continued MMI treatment (10 mg/day), with TRAb levels remaining elevated at 7.3 U/L.

### Patient 2

A 75-year-old female was referred to the Endocrinology Unit for a multinodular goiter. Ultrasound showed an enlarged thyroid with a prominent right lobe that extended into the jugular area, isoechoic with a nodular structure and normal intraglandular vascularization. The largest nodule, located in the mid-to-lower portion of the right lobe, appeared isoechoic, predominantly solid, peripherally vascularized, and measured 21 × 25.5 × 42.9 mm (estimated volume: 11.9 mL).

Due to progressive nodule growth and compressive symptoms, TA was proposed. Pre-procedure blood tests revealed TSH of 0.62 µUI/mL, negative calcitonin, negative Tg antibodies, and positive TPO antibodies (11.71 UI/mL, NR: <5.61). TRAb were not assessed. A repeat FNAC confirmed a benign nodule (Bethesda II/TIR2). No cytological features suggestive of preexisting thyroid autoimmunity were observed.

RFA of the right thyroid nodule was performed. After 1 month, ultrasound revealed a reduction in volume to 7.92 mL (VRR: 34%). The patient reported no symptoms, and no biochemical reassessment was performed at that time.

After 6 months, the nodule had slightly decreased to 7.43 mL (VRR: 38%) with absent vascularization. Biochemical testing showed a low TSH (0.1 µUI/mL) with FT4 of 12.9 ng/L (9.3–17) and FT3 of 3.6 pg/mL (2–4.4). The patient was asymptomatic, and the initiation of antithyroid therapy was postponed.

Four months later, the patient experienced tachycardia. Blood tests confirmed hyperthyroidism with suppressed TSH (<0.001 µUI/mL), FT4 in the upper normal range, mildly elevated FT3 at 3.99 ng/L (NR: 1.71–3.71), and elevated TRAb at 2.1 U/L (NR: <1.5), consistent with GD. MMI (5 mg daily) was initiated. At the most recent follow-up, 20 months after the RFA procedure, the patient was euthyroid, but TRAb remained slightly elevated (TRAb 1.7 U/L; NR: <1.22). No orbitopathy was observed.

### Patient 3

A 39-year-old female with a known history of Hashimoto’s thyroiditis (diagnosed in 2014 with positive Tg and TPO antibodies) was referred to the Endocrinology Unit.

Ultrasound revealed an enlarged thyroid with multiple hypoechoic areas and mildly increased vascularization. There was a dominant isthmic nodule, isoechoic and poorly vascularized, measuring 24.5 × 47.7 × 39.4 mm (estimated volume: 24.3 mL). A prior FNAC in 2019 was non-diagnostic (Bethesda I/TIR1), and ethanol ablation was performed. A repeat FNAC in 2022 confirmed benign features (Bethesda II/TIR2). No cytological features suggestive of preexisting thyroid autoimmunity were observed.

The patient underwent RFA of the isthmic nodule. Two years later, the nodule measured (21 × 44.3 × 38.1 mm, estimated volume: 17 mL), and a second TA by MWA was performed in November 2024. Baseline biochemical tests showed TSH 0.81 µUI/mL, negative calcitonin, markedly elevated TPOAb (847 IU/mL; NR: <5.61) and positive TgAb (39.57 IU/mL; NR: <4.11). TRAb were not assessed.

In early February 2025, during the 13th week of a confirmed pregnancy (singleton), the patient presented with suppressed TSH, elevated FT3 (6.49 ng/L; NR: 1.71–3.71), elevated FT4 (2.12 ng/dL; NR: 0.7–1.48), and TRAb at 1.6 U/L (NR: <1.5).

No symptoms suggesting hyperemesis gravidarum were referred. Increased vascularization on thyroid Doppler-ultrasound supported the diagnosis of GD, and propylthiouracil (PTU) 100 mg daily was initiated.

By mid-February 2025, TSH remained suppressed, but FT3 and FT4 levels showed a decreasing trend (FT3: 4.66 ng/L; FT4: 1.66 ng/dL) under continued PTU therapy at 100 mg/day. Over the following weeks, PTU was gradually tapered and discontinued by the 20th week of gestation (mid-March 2025).

At the 24th week of gestation, 1 month after discontinuation of antithyroid therapy, thyroid function tests confirmed biochemical stability, with TSH at 0.43 µUI/mL, FT3 at 3.2 ng/L, FT4 at 1.06 ng/dL, and TRAb within normal limits (1.4 U/L, NR: < 1.5). At the 31st week, TRAb had further decreased (1.26 U/L; NR: <1.5), and thyroid function remained stable without therapy. The patient subsequently delivered a healthy infant in July 2025. Three months after delivery, laboratory tests showed positive TRAb (1.8 U/L; NR: <1.5). Thyroid function indicated recurrent hyperthyroidism, with suppressed TSH (<0.008 µUI/mL), FT4 at the upper limit of normal (1.43 ng/dL; NR: 0.7–1.48), and mildly elevated FT3 (4.97 ng/L; NR: 1.71–3.71), consistent with a postpartum relapse of GD, and methimazole therapy was initiated.

Clinical and biochemical data of the three patients are summarized in Table 1.

**Table 1:** Clinical and biochemical features of Graves’ disease cases following thyroid nodule ablation.

Parameters	Patient 1	Patient 2	Patient 3	Normal range
Sex	Female	Female	Female	
Age at diagnosis (years)	50	75	39	
Date of procedure	June 2023	July 2023	November 2024	
Pre-procedure				
TSH, μUI/mL	0.68	0.62	0.81	0.35–4.94
TgAb, UI/mL	0.53	0.60	39.57	<4.11
TPOAb, Ul/mL	0.23	11.71	847.65	<5.61
Thyroid volume (mL)	20	21	24.3	
Nodule volume (mL)	12	11.9	17	
Type of procedure	RFA	RFA	MWA	
Total energy delivered (kJ)	35.5	39	6.8	
Energy delivered (kJ/mL)	2.98	3.27	0.4	
Onset of GD after procedure	6 months	10 months	3 months	
Thyroid function at onset of GD				
TSH, μUI/mL	<0.005	<0.001	<0.001	
fT4, ng/L	17.9	1.17	2.12	0.7–1.48
fT3, ng/L	NA	3.99	6.49	1.71–3.71
TRAb (U/L) at diagnosis	7.6	2.1	1.6	<1.5
Ophthalmopathy	No	No	No	
Initiation of therapy after procedure	8 months	10 months	3 months	
Thyrostatic therapy – starting dose				
MMI	20 mg	5 mg		
PTU			100 mg	
Post procedure	August 2024	May 2024	September 2025	
TgAb, UI/mL	0.38	1.46	34.44	<4.11
TPOAb, Ul/mL	0.27	61.04	742.98	<5.61
At last follow-up	June 2025	April 2025	October 2025	
TRAb, U/L	7.3	1.7	1.8	<1.5
Duration of therapy	Ongoing	Ongoing		
PTU (for 6 weeks during pregnancy)			100 mg/day	
MMI (started 3 months postpartum)			5 mg/day	

TSH, thyroid-stimulating hormone; fT4, free thyroxine; fT3, free triiodothyronine; TRAb, TSH receptor antibodies; TgAb, thyroglobulin antibodies; TPOAb, thyroid peroxidase antibodies; GD, Graves’ disease; RFA, radiofrequency ablation; MWA, microwave ablation; MMI, methimazole; PTU, propylthiouracil; NA, not available.

TRAb measurement at the time of GD diagnosis was performed using the Alinity I system (Abbott Laboratories, USA), an automated chemiluminescent microparticle immunoassay (CMIA). The assay has a measurement interval of 1.25–50.00 U/L. The intra-assay coefficients of variation (CVs) ranged from 1.1 to 4.8%, while the intra-laboratory CVs ranged from 1.2 to 5.2%. The analytical sensitivity was defined by a limit of blank (LoB) of 0.38 U/L, a limit of detection (LoD) of 0.70 U/L, and a limit of quantitation (LoQ) of 1.26 U/L. According to the manufacturer’s reference range, TRAb values < 1.5 U/L are considered negative.

## Discussion

We describe three cases of new-onset GD occurring 3–10 months after RFA or MWA among more than 500 patients treated at our center. All patients were female, had cytologically benign nodules, and presented with suppressed TSH, elevated thyroid hormones, TRAb positivity, and increased thyroid vascularization at Doppler ultrasound.

All patients were clinically and biochemically euthyroid before TA. In line with current recommendations, TRAb were not measured before TA. Although the lack of pre-TA TRAb measurement could represent a limitation of the present study, it should be highlighted that systematic evaluation of thyroid autoantibodies, in particular TRAb, before TA is not currently recommended by most recent guidelines/protocols. In general, TRAb routine measurement in the absence of biochemical and/or clinical suspicion of hyperthyroidism is generally not performed owing to a poor cost/benefit ratio. Moreover, the occasional detection of positive tests for TRAb in biochemically euthyroid patients poses interpretative challenges, as the prognostic value of such a finding remains uncertain. Indeed, according to a recent study (8) evaluating euthyroid patients with positive TRAb, i) a very low prevalence (<1%) of positive tests for TRAb is observed in euthyroid patients; ii) only 6 of the 47 subjects (12.8%) developed GD throughout a median follow-up time of 18.3 months; in most patients, TRAb turned negative over time.

At difference with TRAb, TPOAb, and TgAb were measured before and after TA, with two patients showing pre-TA positive tests for TPOAb and/or TgAb without relevant changes after TA which is in agreement with previous evidence indicating that TgAb and TPOAb titers usually remain stable after TA of benign thyroid nodules, with only occasional transient fluctuations, particularly in patients with high baseline titers (9).

TA under ultrasound guidance is a well-established, minimally invasive treatment for benign thyroid nodules, offering a nonsurgical alternative that effectively reduces nodule volume while preserving surrounding thyroid parenchyma. The development of GD following TA was rarely reported in the literature, either reported from series of patients (*n* = 2) treated by TA and/or case reports (*n* = 2), and remains poorly understood. Wang *et al.* (4) investigated thyroid dysfunction following TA, reporting that 36% of patients developed subclinical thyrotoxicosis 1 week after the procedure, likely due to transient thyroid hormone release following TA. At 12 months, persistent thyroid dysfunction was observed in 12% of patients, with 6% developing subclinical hypothyroidism, 4% subclinical thyrotoxicosis, and 1% overt thyrotoxicosis. GD was reported in only one patient after 6 months from the procedure. Similarly, Ben Hamou *et al.* (5) described one patient who developed GD post-TA. This patient was TRAb-negative before the procedure, turned positive for TRAb within the first month following the procedure, and required antithyroid therapy. The first case report of GD following RFA was published in 2023 by McAninch *et al.* (6), who reported the case history of a male patient developing GD 8 weeks after TA procedure. However, his pre-procedural TSH level was 0.3 mUI/L, raising the possibility that underlying thyroid dysfunction may have already been present before the intervention. This could suggest that rather than being a direct trigger, TA may have accelerated an existing autoimmune process or unmasked subclinical GD. Moreover, a recently published case report by Gu *et al.* (7) described a 43-year-old male who developed GD approximately 6 months after undergoing microwave ablation for benign thyroid nodules. Notably, the patient had pre-existing autoimmune thyroiditis before the procedure. Post-ablation, the patient developed hyperthyroidism with positive tests for TRAb, supporting the hypothesis that thermal injury may exacerbate latent autoimmune conditions or trigger new autoimmune activation. The authors also speculated on the role of autonomic nervous system overstimulation and thermal damage to surrounding tissues as potential contributors to the development of GD following ablation therapy. The exact mechanisms underlying the development of GD following TA remain unclear. However, it could be hypothesized that the destructive process induced by TA may trigger autoimmune activation.

Although no mechanistic conclusions as to the link between TA and subsequent occurrence of GD are available, previous research on other thyroid interventions suggests a possible connection between thyroid trauma, antigen release, and immune system activation. Indeed, studies on radioiodine therapy (131I) have shown that thyroid damage can promote thyroid autoimmunity. In the study by Lindgren *et al.* (10), it was reported that a previously TRAb-negative patient with toxic adenoma developed TRAb, TPOAb, and TgAb within 3–4 months after treatment with radioiodine. Another study by Laurberg *et al.* (11) investigated the effect of radioiodine treatment on TRAb, TPOAb, and TgAb, showing that all of the three antibodies increased after 3 months, even if not in all patients. In addition, thyroid manipulation during parathyroidectomy (12) and external head and neck irradiation (13) has been linked to autoimmune thyroid diseases. Similarly, percutaneous ethanol ablation has been associated with Graves’ ophthalmopathy (GO) (14).

Moreover, there are reports of GD occurrence following subacute thyroiditis (SAT), with the interval between the onset of SAT and the diagnosis of GD ranging from 1 to 8 months (15, 16, 17). These reports suggest various mechanisms leading to GD after SAT, including immune system stress in predisposed patients, release or expression of autoantigens in susceptible individuals, and/or genetic susceptibility to the disease. Bartalena *et al.* (15) reported a case history of a patient with no personal or familial history of thyroid disorders, and showing negative tests for thyroid autoantibodies at the diagnosis of SAT, who developed typical GD 4 months later.

The common denominator among these evidences is that thyroid tissue damage, caused by ethanol injection, external irradiation, radioiodine therapy, or thyroid manipulation during surgery, can release thyroid antigens including TSHR protein, even in the absence of preexisting antithyroid antibodies. This antigenic release may initiate an autoimmune response, potentially leading to GD.

All of the above findings support the idea that thyroid injury, regardless of the method, may act as a trigger for autoimmune processes. Thus, TA could induce GD through mechanisms such as tissue necrosis and subsequent inflammation leading to thyroid antigen release. If thyroid destruction and autoantigen release are common features of various thyroid-destructive processes (such as SAT, TA, and radioiodine therapy), a key question arises: why is subsequent GD-related hyperthyroidism so rare? The answer likely lies in genetic susceptibility to thyroid autoimmunity. Not all individuals who experience thyroid injury develop GD, suggesting that genetic factors play a crucial role.

## Conclusion

In conclusion, although TA for benign thyroid nodules is generally safe and well tolerated, clinicians should be aware of the rare possibility of triggering autoimmune hyperthyroidism, such as GD.

This appears to be of potential clinical relevance, as it underscores the importance of distinguishing transient post-ablation thyrotoxicosis from the rarer onset of GD, to ensure timely initiation of antithyroid therapy when appropriate.

The results reported here (3 GD out of 500 TA procedures, performed) appear in line with a small risk for developing GD, which would not justify systematic screening for TRAb levels in all patients addressed to TA. On the other hand, pre-TA measurements of TgAb and TPOAb appear to be of limited clinical relevance in predicting post-TA thyroid dysfunctions.

## Declaration of interest

The authors declare that there is no conflict of interest that could be perceived as prejudicing the impartiality of the work reported.

## Funding

FM and FC were partially supported by the National Recovery and Resilience Plan (NRRP), Mission 4 component 2 investment 1.3 – call for proposals No. 341 of 15 March 2022 of the Italian Ministry of University and Research funded by the European Union – NextGenerationEU; Award Number: project code PE00000003, concession decree No. 1550 of 11 October 2022 adopted by the Italian Ministry of University and Research, CUP D93C22000890001, Project title ‘ON Foods – Research and innovation network on food and nutrition Sustainability, Safety and Security – Working ON Foods’. This work was partially supported by the ‘Ricerca Corrente’ funding scheme of the Ministry of Health Italy.

## Patient consent

Written informed consent was obtained from the patient for publication of the submitted article and accompanying images.

## Author contribution statement

MT, BG, and IC wrote the manuscript and collected case data. FM, FC, LC, SC, and MR reviewed it critically. All authors approved the version to be published.

## Statement of ethics

All procedures were in accordance with the ethical standards of the institutional research committee and with the 1964 Helsinki Declaration and its later amendments. Written informed consent for publication of their clinical details was obtained from all patients included in this case series. The consent was obtained by MT.
